# Evaluation of Ground and Aerial Ultra-Low Volume Applications Using ReMoa Tri Against Deltamethrin-Resistant *Aedes aegypti* from Collier County, Florida

**DOI:** 10.3390/tropicalmed10050119

**Published:** 2025-04-26

**Authors:** Decyo McDuffie, Sara Kacinskas, Suzanne Li, Casey Parker-Crockett, Keira J. Lucas

**Affiliations:** 1 Collier Mosquito Control District, Naples, FL 34104, USA; dmcduffie@cmcd.org (D.M.); skacinskas@cmcd.org (S.K.); sli@cmcd.org (S.L.); 2 Azelis, Lake Mary, FL 32746, USA; ab73@cdc.gov; 3 Division of Vector-Borne Diseases, Centers for Disease Control and Prevention, Fort Collins, CO 80521, USA

**Keywords:** macrocyclic lactone, abamectin, fenpropathrin, deltamethrin, *Aedes aegypti*, insecticide resistance

## Abstract

New intervention methods and product formulations are needed to better control pyrethroid-resistant *Aedes aegypti* populations and mitigate the risk of mosquito-borne disease. ReMoa Tri is a novel adulticidal space spray that utilizes a different mode of action than the commonly used adulticides: pyrethroids and organophosphates. As a triple-action space spray, ReMoa Tri combines three components: Fenpropathrin, a mixed-type I/II pyrethroid; abamectin, a macrocyclic lactone; and C8910, a patented fatty acid chain. Prior studies performed by Collier Mosquito Control District showed that ReMoa Tri is effective at controlling type I pyrethroid-resistant *Ae. aegypti* mosquitoes. To further validate these results and the performance of ReMoa Tri, we conducted a semi-field evaluation using ground and aerial ULV (ultra-low volume) applications with field-caught deltamethrin-resistant *Ae. aegypti* and a susceptible *Ae. aegypti* laboratory strain. Ground evaluations tested ReMoa Tri and a type II pyrethroid-based product, DeltaGard. While ReMoa Tri was equally effective against Collier’s deltamethrin-resistant *Ae. aegypti* and the susceptible laboratory strain, DeltaGard was effective against both strains, with reduced efficacy at farther distances. Similarly, aerial evaluations also showed that ReMoa Tri was equally effective against Collier’s deltamethrin-resistant *Ae. aegypti* strain and susceptible laboratory strain. This study further confirms ReMoa Tri’s potential as an effective alternative to pyrethroid-based adulticides, both in ground and aerial applications, for managing pyrethroid-resistant *Ae. aegypti*.

## 1. Introduction

Collier County, Florida, is a prominent destination for both tourists and new residents. The region’s increasing population growth and ongoing urban development contribute to the expanded distribution potential of the yellow fever mosquito, *Aedes aegypti* (L.) [1,2]. This species is a known vector for several significant arboviruses, including dengue, chikungunya, yellow fever, and Zika. Florida has historically experienced outbreaks and the local transmission of some of these viruses [3,4,5] and has more recently seen an influx of travel-related and local transmission of dengue virus [6]. To mitigate the risk of disease transmission and effectively manage *Ae. aegypti* populations, implementing an integrated mosquito management strategy is essential. This comprehensive approach typically involves rotating chemical classes and using both adulticides and larvicides to target multiple mosquito life stages, thereby maximizing the control efficacy and managing resistance [7,8].

Controlling the adult *Ae. aegypti* can present significant challenges. In the United States, the two primary classes of adulticides used for mosquito control are pyrethroids and pyrethrins, and organophosphates. The Insecticide Resistance Action Committee (IRAC) classifies pyrethroids and pyrethrins (IRAC Group 3A) as voltage-gated sodium channel (VGSC) modulators, while organophosphates (IRAC Group 1B) function as acetylcholinesterase inhibitors [9]. With only two available modes of action for adult mosquito control, implementing true insecticide rotation strategies is not feasible—particularly when resistance compromises the effectiveness of one or both classes. Unfortunately, pyrethroid resistance in *Ae. aegypti* populations has been extensively documented across Florida [10,11,12,13,14,15,16,17,18]. More recently, resistance to organophosphates has been detected in *Ae. aegypti* populations throughout Florida [11].

In Collier County, *Ae. aegypti* populations have shown strong resistance to pyrethroid-based products due to certain resistance mechanisms: knockdown resistance (*kdr*) [10] and metabolic resistance [12]. Pyrethroids prolong the opening of sodium channels, which may cause excitatory paralysis before death occurs, causing a “knockdown” effect. This resistance mechanism is due to point mutations in the VGSC gene that decreases the mosquito’s sensitivity to the insecticide. Collier County *Ae. aegypti* have been shown to carry a high frequency of *kdr* alleles, which include the conversion of valine (V) to isoleucine (I) at position 1016 (V1016I) and phenylalanine (F) to cysteine (C) at position 1534 (F1534C) [10]. Metabolic detoxification enzymes degrade the insecticide and limit or inhibit the insecticide’s toxicity to the mosquito. These enzymes come from multigene families that include oxidases, esterases, and glutathione S-transferases [19]. Metabolic enzyme-based resistance in *Ae. aegypti* populations in Collier County has been shown to be attributed to esterase activity [12]. Further, organophosphate resistance has been identified in *Ae. aegypti* populations in Collier County [11,18]. With the high levels of resistance to these conventional pesticides, a need is created for new control chemistries to be added to integrated mosquito management plans.

ReMoa Tri^®^ (4% fenpropathrin; 1.5% abamectin; 0.33% octanoic acid; 0.33% nonanoic acid; and 0.33% decanoic acid) (Valent Biosciences, Libertyville, IL, USA) is a new adulticidal space spray that offers a third class of adulticide for mosquito control. ReMoa Tri is a triple-action spray that combines three active ingredients: abamectin (avermectin B1), the mixed type I/type II pyrethroid Fenpropathrin, and a blend of fatty acids known as C8, C9, and C10 (collectively called C8910). Abamectin (IRAC group 6) is a macrocyclic lactone that is produced by a soil bacterium known as *Streptomyces avermitilis*. C8910 is a patented fatty acid chain comprised of saturated fatty acids that fall under the IRAC group UNE, with an unknown mode of action.

Ground-based applications of ReMoa Tri have demonstrated effectiveness in controlling *Ae. aegypti* populations exhibiting resistance to type I pyrethroids and developing resistance to naled under semi-field conditions [18]. Additionally, ReMoa Tri has proven effective in managing *Culex quinquefasciatus* populations with documented pyrethroid resistance in both Collier County [18] and Miami-Dade County [20].

While resistance and field efficacy against type I pyrethroids has been extensively studied in Collier County, the susceptibility of *Ae. aegypti* to type II pyrethroids remains unexamined. Type II pyrethroids are believed to be more effective than type I pyrethroids against resistant mosquito populations due to their ability to prolong the opening of voltage-gated sodium channels [21]. This extended opening increases the duration for active ingredients to penetrate the mosquito’s membrane, thereby enhancing insecticidal efficacy.

This study aimed to understand the effectiveness of ground-based, ultra-low volume (ULV) applications of DeltaGard^TM^ (2% deltamethrin) (ENVU Environmental Science, Cary, NC, USA) and ReMoa Tri against *Ae. aegypti* resistant to deltamethrin. Additionally, we evaluated the efficacy of aerial ULV applications of ReMoa Tri against deltamethrin-resistant *Ae. aegypti*. Prior to this study, no published research had examined the use of ReMoa Tri for aerial ULV adulticiding. These aerial trials represent the first evaluation of ReMoa Tri’s efficacy when applied via aerial ULV methods. Here, we present our findings on the effectiveness of both ground and aerial applications of ReMoa Tri against deltamethrin-resistant *Ae. aegypti* under semi-field conditions.

## 2. Methods

### 2.1. Mosquito Collections and Rearing

The field strain of *Ae. aegypti* (Collier-*Ae. aegypti*) was collected by placing ovitraps, containing seed germination paper and water, in Golden Gate City for oviposition for 5 days (within a 1 mi (1.6 km) radius of 26.18363° N, −81.70190° W) in Naples, Florida. Eggs were hatched and larvae were reared through an F2 population in the insectary at 28 °C (82.4 °F), 80% relative humidity, and a constant 14-hour-light:10-hour-dark cycle. Larvae were provided with a diet of equal parts dog chow, lactalbumin, and brewer’s yeast. Adult mosquitoes were supplied with 10% (wt/vol) sucrose solution ad libitum. Susceptible Benzon Research 1994 *Ae. aegypti* (Benzon Research, Carlisle, PA, USA) (susceptible-*Ae. aegypti*) were received as 3–5 day old adults and kept in the insectary regulated as described above.

### 2.2. Insecticide Susceptibility Tests

Three technical replicates of the CDC bottle bioassay were conducted with approximately 15–20 adult female susceptible-*Ae. aegypti* and Collier-*Ae. aegypti*, which were exposed to the CDC diagnostic dose of technical grade deltamethrin at 0.75 μg/mL [22], which was diluted with acetone to deliver the CDC diagnostic dose in 1 mL of solution. The mosquitoes were also exposed to DeltaGard, which was diluted in acetone to yield the equivalent CDC diagnostic dose of active ingredient. Wheaton media bottles (250 mL) were coated with either one of the deltamethrin test solutions or an acetone control. Bottles were dried void of light for 2 h and used immediately for the assay. The knockdown was recorded every 15 min for 2 h. The knockdown was recorded if the mosquitoes could no longer stand, behaved erratically, or could not maintain flight. The percent mortality was calculated and corrected using Abbott’s formula [23]. The resistance status was defined based on the World Health Organization guidelines [24].

### 2.3. Ground-Based Field Evaluation of ReMoa Tri and DeltaGard

The test location was a large, open field located at the Collier County Fairgrounds (26.30571° N, −81.58814° W). The test plot was a 3 × 3 grid design with three rows of three sampling stations positioned at 30.48 m (100 ft), 60.96 m (200 ft), and 91.44 m (300 ft) downwind from the ULV application line. Sampling station replicates (technical replicates) at each position were placed 30.48 m (100 ft) apart perpendicularly to the ULV application to create the grid. Each sampling station included a stand that held two cages (one cage of each test strain). Standard bioassay cages (15.2 cm diameter × 3.8 cm) were affixed to a rotating arm with a windvane to orient the cages perpendicularly to the prevailing wind direction. Each station also included a rotating impinger (Leading Edge, Port Orange, FL, USA) with 3 mm (0.12 in) Teflon-coated acrylic rods for droplet collection.

The field cage trials occurred on 16 October 2024. On the day of the trial, 10–20 3–5-day-old adult mosquitoes of the appropriate strains were aspirated into each field cage. A total of 162 susceptible-*Ae. aegypti* and 142 Collier-*Ae. aegypti* were exposed to ReMoa Tri, 131 susceptible-*Ae. aegypti* and 124 Collier-*Ae. aegypti* were exposed to DeltaGard, and 40 each were used for the non-treated controls. Each cage was provided with cotton rounds soaked in a 10% sucrose solution and stored at 28 °C prior to the transport to the test location. At the test location, three control cages of each strain were hung for 30 min and taken down prior to the start of the application. Mortality was checked prior to the application. A weather station tracking humidity, windspeed, wind direction, and temperature at both 9.14 m (30 ft) and 1.5 m (5 ft) from ground level was placed at the application site to monitor the atmospheric conditions during the trial. The trials were conducted under atmospheric conditions when wind velocity was 1.6–16 kph (1–10 mph) and a temperature inversion existed. The treatment parameters for each application are described in Table 1.

The drive line was set 30.48 m (100 ft) downwind from the first line of sampling stations. The ULV sprayer was turned on 60.96 m (200 ft) before the first row of sampling stations and turned off 60.96 m (200 ft) after the last row of sampling stations to ensure adequate coverage. ReMoa Tri was delivered at a slightly above mid-label application rate for the resistant mosquitoes of 0.83 oz/acre (fenpropathrin: 0.00195 lb/acre; abamectin: 0.00071 lb/acre; C8910: 0.00052 lb/acre) and DeltaGard was delivered at a high-label application rate of 0.95 oz/acre (deltamethrin: 0.00126 lb/acre). The DynaJet L30 ULV Sprayer (Curtis Dyna-Fog, Jackson, GA, USA) was calibrated prior to each application to deliver the target application rate for each product at a speed of 16 kph (10 mph) with an anticipated swath of 91.44 m (300 ft).

The caged mosquitoes and rotating, Teflon-coated acrylic rods were set immediately prior to application and collected 20 min post-application. Upon collection, the caged mosquitoes were immediately transferred to holding containers on site and brought back to the laboratory for evaluation. The mosquitoes were provided with a 10% sucrose solution. The mortality was recorded at 24 h and 48 h post-application. The mortality was recorded if the mosquitoes could no longer stand, behaved erratically, or could not maintain flight. The corrected percent mortality was calculated using Abbott’s formula [23]. A graphical analysis and the determination of statistical significance using multiple unpaired *t*-tests was performed using GraphPad Prism version 9.0.0 for macOS (GraphPad Software, Boston, MA, USA). The Teflon-coated acrylic rods from each sampling station were analyzed by the A-Drop™ (Valent Biosciences, Libertyville, IL, USA) droplet analysis program.

### 2.4. Aerial Evaluation of ReMoa Tri

The test location, test plot, and sampling location were set up as described above. The field cage trials occurred on 16 August 2024 and consisted of two 3 × 3 test plots approximately 200 m (656 ft) apart. On the night before the trial, 10–20 3–5-day-old adult mosquitoes of the appropriate strains were aspirated into each field cage. A total of 138 susceptible-*Ae. aegypti* and 141 Collier-*Ae. aegypti* were exposed to ReMoa Tri and 43 susceptible-*Ae. aegypti* and 46 Collier-*Ae. aegypti* were used for the non-treated controls for test plot 1. A total of 133 susceptible-*Ae. aegypti* and 140 Collier-*Ae. aegypti* were exposed to ReMoa Tri and 49 susceptible-*Ae. aegypti* and 48 Collier-*Ae. aegypti* were used for the non-treated controls for test plot 2. Each cage was provided with cotton rounds soaked in a 10% sucrose solution and stored at 28 °C (82.4 °F) prior to the transport to the test location the following morning. At the test location, three control cages of each strain were hung for 30 min and taken down prior to the start of the application. A weather station tracking humidity, windspeed, wind direction, and temperature at 1.5 m (5 ft) from ground level was placed at the application site to monitor the atmospheric conditions during the trial. In addition, on-board meteorology was used to determine the temperature at altitude (76.2 m [250 ft]), and the windspeed and direction at altitude were determined using a weather-tracking station located on a nearby tower at altitude. The trials were only conducted during stable atmospheric conditions when the wind velocity at altitude was 4.8–16 kph (3–10 mph).

ReMoa Tri was delivered at 0.83 oz/acre (fenpropathrin: 0.00195 lb/acre; abamectin: 0.00071 lb/acre; C8910: 0.00052 lb/acre) using a Bell 407 helicopter (Bell Textron, Fort Worth, TX, USA) outfitted with a spray system consisting of a boom on each side of the aircraft. Each boom was equipped with a Micronair^®^ AU6539 electric atomizer (Micron Group, Bromyard, Herefordshire, UK). The application was made to a 1281.2 acre spray block using an anticipated swath of 243.84 m (800 ft) at a speed of 148 kph (80 kt) and an altitude of 76.2 m (250 ft). The flight planning and navigation was performed using AgMission (AgNav GPS Precision Navigation, Barrie, ON, Canada). A previously established AgNav-developed drift model for Merus 3.0 was used to account for drift, with the assumption that ReMoa Tri (density = 0.915 g/cm^3^ [7.64 lbs/gal]) would behave similarly to Merus 3.0 (density = 0.874 g/cm^3^ [7.29 lbs/gal]) when applied at the same altitude and by the same aircraft.

The caged mosquitoes and rotating, Teflon-coated acrylic rods were collected 60 min post-application. Upon collection, the caged mosquitoes were immediately transferred to holding containers on site and brought back to the laboratory for evaluation. The mosquitoes were provided with a 10% sucrose solution. The mortality was recorded at 24 h and 48 h post-application, as described above. The graphical analysis and statistical significance were determined as described above. The Teflon-coated acrylic rods from each sampling station were analyzed as described above.

## 3. Results

### 3.1. Susceptibility of Collier-Aedes aegypti to Deltamethrin and Deltagard

**Figure 1:**
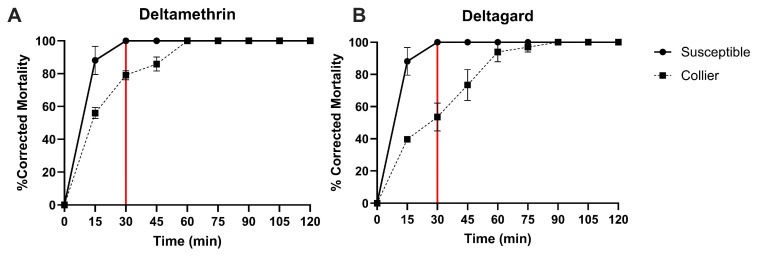
Centers for Disease Control and Prevention (CDC) bottle bioassays for susceptible-*Aedes aegypti* (solid line) and F2 Collier-*Ae. aegypti* (dashed line). CDC bottle bioassays using technical-grade deltamethrin (**A**) and formulated Deltagard (**B**) with the diagnostic dose (0.75 ug deltamethrin/bottle). The solid vertical red line indicates the published threshold for the CDC diagnostic dose of the susceptible *Ae. aegypti* REX colony. Data represent 3 technical replicates and are shown as mean ± SEM.

The Collier-*Ae. aegypti* used in the field trials and the susceptible-*Ae. aegypti* were subjected to a CDC bottle bioassay, as described in the methods section. The susceptible-*Ae. aegypti* displayed 100% mortality at the CDC diagnostic time for deltamethrin (15 min) when exposed to technical-grade deltamethrin and formulated DeltaGard (Figure 1A,B). The Collier-*Ae. aegypti* strain exhibited resistance to deltamethrin, with 79.13 ± 2.86% (mortality ± Standard Error of Mean [SEM]) mortality for technical-grade deltamethrin and 53.55 ± 8.68% mortality for formulated DeltaGard at the 30 min timepoint (Figure 1A,B). After 2 h of exposure, the mortality reached 100% for both the technical-grade and formulated products (Figure 1A,B). These data signify that the Collier-*Ae. aegypti* used in the field trials are resistant to the type II pyrethroid, deltamethrin.

### 3.2. Ground Applications of ReMoa Tri and DeltaGard Targeting Collier-Aedes aegypti

The ReMoa Tri application was conducted in stable conditions with 8.87 kph (5.51 mph) winds from the north, with an ambient temperature of 21.33 °C (70.4 °F). The DeltaGard application was conducted with a higher windspeed, namely 10.7 kph (6.65 mph) winds from the north, with an ambient temperature of 22.74 °C (72.93 °F). The conditions during the DeltaGard application were slightly unstable, with wind gusts of up to 20.44 kph (12.7 mph). The treatment parameters of each application are described in Table 1.

**Figure 2:**
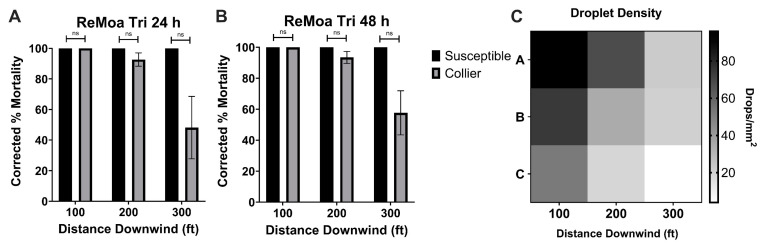
Ground-based, semi-field cage trials using ReMoa Tri against susceptible-*Ae. aegypti* and Collier-*Ae. aegypti*. (**A**,**B**) *Ae. aegypti* corrected %mortality at 24 h (**A**) and 48 h (**B**) post-application of ReMoa Tri. Data represent 3 technical replicates and are shown as mean ± SEM. Multiple unpaired *t*-tests were performed to indicate statistical significance: ns = not significant. (**C**) Droplet density (mm^2^) for ReMoa Tri applications at each sampling station. Rows A, B, and C represent technical replicates.

ReMoa Tri was effective against the susceptible-*Ae. aegypti*, with 100% mortality at each distance downwind of the spray origin at 24 h (Figure 2A) and 48 h (Figure 2B) post-application. By 24 h post-application, the Collier-*Ae. aegypti* reached 100%, 92.65 ± 4.33%, and 48.13 ± 20.43% mortality at 30.48, 60.96, and 91.44 m, respectively (Figure 2A). By 48 h post-application, increased effectiveness was observed, with the Collier-*Ae. aegypti* reaching 100%, 93.40 ± 3.88%, and 57.63 ± 14.27% mortality at 30.48, 60.96, and 91.44 m, respectively (Figure 2B). No significant difference in effectiveness was observed between the susceptible-*Ae. aegypti* and the Collier-*Ae. aegypti*, indicating that ReMoa Tri was equally effective at knocking down deltamethrin-resistant *Ae. aegypti* as susceptible-*Ae. aegypti*. At the 91.44 m station, the mosquito mortality decreased; however, no significant difference was observed between the tested strains. The reduction in mortality was likely attributed to a decreased droplet density at this distance (Figure 2C). Together, these results indicate ReMoa Tri is an effective space spray for targeting both susceptible and deltamethrin-resistant *Ae. aegypti.* The average volume median diameter (VMD) was 23.1 um.

**Figure 3:**
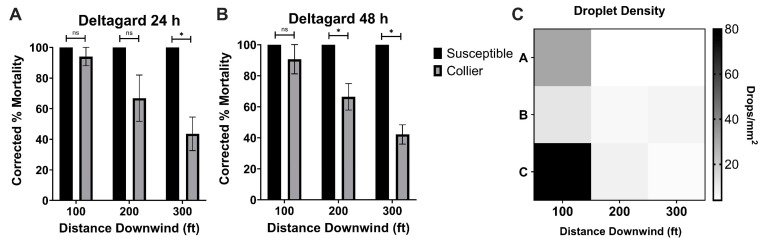
Ground-based, semi-field cage trials using Deltagard against susceptible-*Ae. aegypti* and Collier-*Ae. aegypti*. (**A**,**B**) *Ae. aegypti* corrected %mortality at 24 h (**A**) and 48 h (**B**) post-application of Deltagard. Data represent 3 technical replicates and are shown as mean ± SEM. Multiple unpaired *t*-tests were performed to indicate statistical significance: ns = not significant; * *p* < 0.05. (**C**) Droplet density (mm^2^) for DeltaGard applications at each sampling station. Rows A, B, and C represent technical replicates.

DeltaGard was also effective against the susceptible-*Ae. aegypti*, with 100% mortality at each distance downwind of the spray origin at 24 h (Figure 3A) and 48 h (Figure 3B) post-application. DeltaGard displayed reduced effectiveness against the Collier-*Ae. aegypti*, with 94.01 ± 5.99%, 66.84 ± 15.12%, and 43.55 ± 10.97% mortality at 30.48, 60.96, and 91.44 m, respectively, at 24 h post-application (Figure 3A). However, the Collier-*Ae. aegypti* only displayed significantly reduced mortality compared to the susceptible-*Aedes aegypti* at the 91.44 m (*p* = 0.0203, df = 4) sampling station at 24 h post-application. A similar trend was observed for the 48 h post-application time point, with the Collier-*Ae. aegypti* displaying significantly reduced mortality compared to the susceptible-*Ae. aegypti* at the 60.96 m (*p* = 0.0339, df = 4) and 91.44 m (*p* = 0.002238, df = 4) sampling stations at 48 h post-application (Figure 3B). This was likely due to the recovery that was observed, indicating some knockdown resistance, at the 200 A-C and 300 B-C cages for the Collier-*Ae. aegypti*. These results suggest that DeltaGard is an effective space spray when targeting susceptible *Ae. aegypti* and potentially deltamethrin-resistant *Ae. aegypti* given a consistent dose across the spray line. Average VMD was 21.4 um.

### 3.3. Aerial Applications of ReMoa Tri Targeting Collier-Aedes aegypti

**Figure 4:**
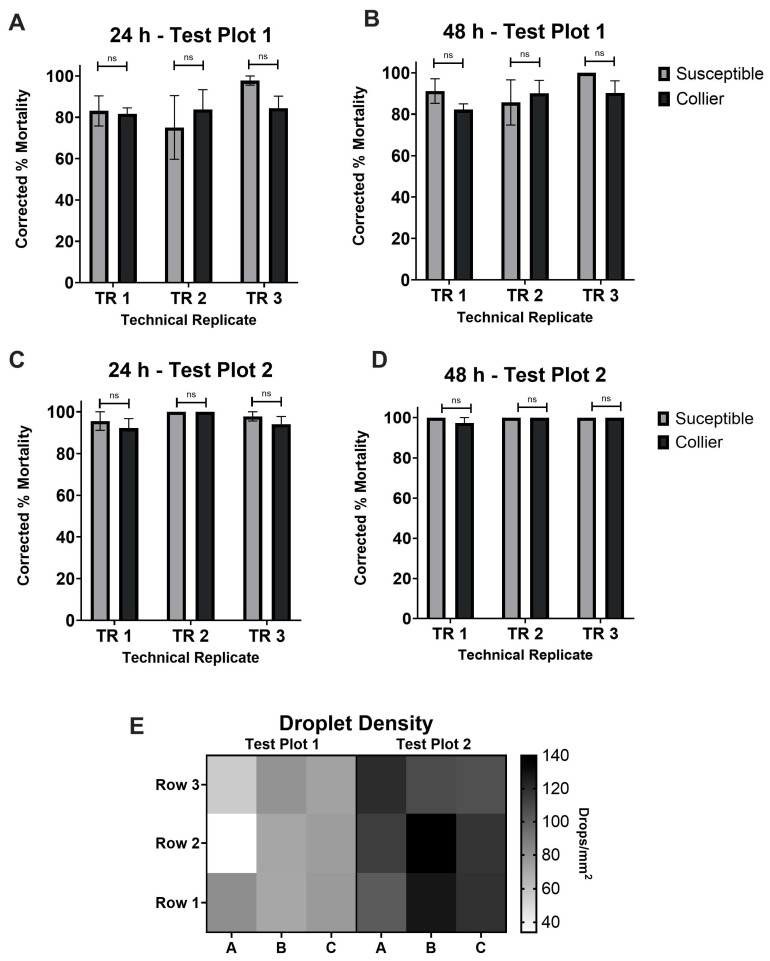
Aerial, semi-field cage trials using ReMoa Tri against susceptible-*Ae. aegypti* and Collier-*Ae. aegypti*. (**A**,**B**) *Ae. aegypti* corrected %mortality at 24 h (**A**) and 48 h (**B**) post-application of ReMoa Tri for test plot 1. (**C**,**D**) *Ae. aegypti* corrected %mortality at 24 h (**C**) and 48 h (**D**) post-application of ReMoa Tri for test plot 2. (**A**–**D**) Data represent 3 technical replicates and are shown as mean ± SEM. Multiple unpaired *t*-tests were performed to indicate statistical significance: ns = not significant. (**E**) Droplet density (mm^2^) for DeltaGard applications at each sampling station. Columns A, B, and C represent technical replicates for each test plot.

The aerial application was conducted under stable ground conditions of 5.5 kph (3.45 mph) winds from the southeast, with an ambient temperature of 26 °C (78.8 °F). The conditions at altitude were also stable, with 12.96 kph (8.05 mph; 7 kt) winds from the southeast and an ambient temperature of 24 °C (75.2 °F). The treatment parameters for the application are described in Table 1. The aerial, semi-field cage trail for ReMoa Tri displayed similar results to the ground trails. In the first test plot, ReMoa Tri was equally as effective against the Collier-*Ae. aegypti* and the susceptible-*Ae. aegypti*, with no significant differences between the two at 24 h (Figure 4A) and 48 h (Figure 4B) post-application. By 24 h post-application, the susceptible-*Ae. aegypti* ranged from 83.10 ± 7.27% to 97.78 ± 2.22% mortality between the three technical replicates, while the Collier-*Ae. aegypti* displayed similar mortality at 81.67 ± 2.92% to 84.44 ± 5.8% (Figure 4A). By 48 h post-application, increased effectiveness was observed, with the susceptible-*Ae. aegypti* reaching 85.67 ± 10.92% to 100% mortality, while the Collier-*Ae. aegypti* reached 82.22 ± 2.80% to 90.32 ± 5.82% effectiveness between the three technical replicates (Figure 4B). The average VMD was 29 um in test plot 1.

The second test plot was substantially more efficacious due to an increased droplet density (Figure 4E). By 24 h post-application, the susceptible-*Ae. aegypti* ranged from 95.56 ± 4.44% to 100% mortality between the three technical replicates, while the Collier-*Ae. aegypti* displayed similar mortality at 92.17 ± 4.56% to 100% (Figure 4C). By 48 h post-application, increased effectiveness was observed, with the susceptible-*Ae. aegypti* reaching 100% mortality and the Collier-*Ae. aegypti* reaching 97.25 ± 2.75% to 100% effectiveness between the three technical replicates (Figure 4D). Together, these results indicate that ReMoa Tri is an effective aerial space spray for targeting both susceptible and deltamethrin-resistant *Ae. aegypti.* The average VMD was 26.3 um in test plot 2.

## 4. Discussion

Insecticide resistance in *Ae. aegypti* presents a significant public health concern, particularly given the increasing transmission of arboviruses by this vector in the United States [6]. In Collier County, *Ae. aegypti* populations have been documented as resistant to both pyrethroids and organophosphates [10,11,12,14,18]. While the presented study focuses exclusively on mosquito populations in Collier County, the trend of insecticide resistance in *Ae. aegypti* is widespread throughout Florida [10,11,12] and, more broadly, the US [25,26]. With only two modes of action previously available for adult mosquito control, implementing adulticide product rotations as recommended by IRAC was not feasible if resistance to either chemical class was detected. However, type II pyrethroids, such as deltamethrin, offer improved field efficacy due to their extended action on target sites. Additionally, novel active ingredients, like abamectin, introduce a new mode of action, providing alternative strategies for managing resistant mosquito populations [18]. Together, these tools can enhance control efforts against resistant *Ae. aegypti* and reduce the risk of arboviral transmission.

This study demonstrated that ground-based ULV applications of DeltaGard, despite containing 2% deltamethrin—a type II pyrethroid—are potentially effective against deltamethrin-resistant *Ae. aegypti* mosquitoes up to 60.96 m from the line of spray. Although mortality was observed in the susceptible strain, a decrease in droplet density was recorded at the 60.96 m and 91.44 m sampling stations (Figure 3B), which may explain the lower mortality rates for deltamethrin-resistant *Ae. aegypti* during the DeltaGard applications at those stations. However, the collection efficiency of the impinger used in the trials may have introduced sampling variability due to elevated wind speeds during the applications, measured at 10.7 kph, with gusts reaching 20.44 kph. High and variable wind speeds can disrupt droplet collection, leading to inconsistent sampling [27]. Alternatively, the uneven delivery of the spray cloud caused by the fluctuating wind conditions could have contributed to the reduced mortality. Despite these challenges in interpreting the data, the finding that DeltaGard is effective despite deltamethrin-resistance in CDC bottle bioassay is similar to previous studies indicating that DeltaGard was effective against deltamethrin-resistant *Ae. aegypti* from Miami-Dade County [8] and Manatee County, Florida [28,29]. In an open field study conducted by Hart and Hare [29] with max label rate DeltaGard against deltamethrin-resistant *Ae. aegypti*, they also observed decreasing mortality in their field population as the distance increased from the line of spray, which aligned with a decreasing droplet density. Additionally, a study evaluating DeltaGard against Californian populations of *Ae. aegypti* showed no significant difference in mortality between the susceptible and the field strain in an open field setting, but significantly lower mortality in the field strain when the trial was conducted in a residential setting [26]. The results from other studies evaluating DeltaGard against *Ae. aegypti* align with the presented studies, indicating that the droplet density significantly impacts the mortality in field populations of *Ae. aegypti*.

Consistent with previous findings [18], the triple-action formulation of ReMoa Tri proved effective against deltamethrin-resistant *Ae. aegypti* using ground ULV applications. The previous study observed 74–97% mortality at 48 h in a field population of *Ae. aegypti* [18], like the presented study for *Ae. aegypti*. Notably, this study represents the first evaluation of ReMoa Tri for aerial ULV applications. The aerial trials demonstrated that ReMoa Tri effectively controlled both susceptible and deltamethrin-resistant *Ae. aegypti*, with high mortality rates observed. While variations in the droplet density influenced efficacy, the results affirm ReMoa Tri’s potential as a valuable tool for large-scale mosquito control operations from the ground and aerially, as well as against multiple vector mosquito species.

## 5. Conclusions

The inclusion of abamectin introduces a novel mode of action to the public health vector control industry, which, alongside fenpropathrin and C8910, may assist in resistance management, mitigate resistance development, and enhance the overall control efficacy. While reports of abamectin resistance in other arthropods has been reported [30,31,32], the incorporation of multiple active ingredients with differing modes of action into a single product employs the multiple attack resistance management strategy [33], theoretically delaying the development of resistance within mosquito populations. Incorporating products like ReMoa Tri into control programs could offer a viable solution to overcoming resistance challenges, particularly when conventional chemistries lose effectiveness. Furthermore, the demonstrated success of aerial applications expands the operational flexibility, allowing for rapid, large-area treatments during peak mosquito activity or outbreak scenarios.

## Figures and Tables

**Table 1:** Environmental parameters during field cage trials.

				Temperature (°C)				
Date	Time	Type	Product	1.524 m	9.144 m/76.2 m	Wind Speed (kph)	Wind Direction	Relative Humidity (%)
16 October 2024	7:24 PM	Ground	DeltaGard	22.74	23.67	10.7	N	65.84
16 October 2024	8:25 PM	Ground	ReMoa Tri	21.33	23.67	8.87	N	63.12
14 August 2024	6:36 AM	Aerial	ReMoa Tri	26.11	24	12.96	SE	82

## Data Availability

The raw data supporting the conclusions of this article will be made available by the authors on request.
